# Its Work, Its Ambitions, and Its Outlook

**Published:** 1917-04-28

**Authors:** 


### THE ROYAL COLLEGE OF BRITISH NURSING.

### ITS WORK, ITS AMBITIONS, AND ITS OUTLOOK.

### IMPORTANT SPEECH BY THE HON. ARTHUR STANLEY, C.B., M.P.

Mr. Stanley gave an address at Stratford, on the 18th inst., in the course of which he exhibited strong proof of his deep interest in the success of the College by remarking that he anticipated he would soon have spoken in the out-patient department of every hospital in the country. Mr. Stanley is always practical, and he impressed upon the meeting and the nursing profession generally that his main object was not to make a speech but to be questioned to the utmost, so that every nurse in the land might fully understand the College scheme and all the great possibilities, safeguards, facilities for educational and practical improvement, protection, strength, and social advantage to which every certificated nurse who joins the College Register will become entitled. Mr. Stanley fixed the minimum for a successful organisation of British certificated nurses at at least 20,000. There are, we believe, some 40,000 certificated, trained British nurses. Already a fourth of them may be regarded as convinced supporters of the College, whose names are either on its Register or will be there shortly. This should mean that the names of the 20,000 certificated British nurses Mr. Stanley fixes as the minimum number for the College should be registered members of it by the spontaneous action of the nurses themselves before its Endowment Fund is completed and lodged in the bank. God helps those who help themselves, and British nurses, thousands of whom have already shown splendid initiative as supporters of the College, should learn to keep busy, and keep busy completing their Registration Papers, registering themselves and recruiting friends for the Register.

Once on the Register Mr. Stanley showed in his speech that a British nurse will have behind her a powerful friend, capable of tiding her over periods of illness, accident, and difficulty, providing she has given some of the best years of her life to the benefit of humanity, and any woman who does this is entitled to any benefit she may receive from the funds which the College may make available, for that is her simple due. Every certificated nurse who immediately applies for Registration with the College will spontaneously help herself and her sisters, through Mr. Stanley and his friends, who are supporters of the College, to build up the Register, to build up the Endowment Fund, and to build up a big Endowment Fund too.

Mr. Stanley justly said that the nursing profession was never held so highly by all classes as it is at present. The one outstanding memory of the Crimean War, thanks to Florence Nightingale's noble work and example, is the nursing profession as we know it to-day. The great nursing profession all over the world was the creation of Florence Nightingale, and Mr. Stanley's hope is for British nursing to carry it another step onwards, and as the outcome of the present war to organise the nursing profession and give the nation and Empire the finest memorial by placing the Royal College of British Nursing upon sure foundations, foundations wide enough and strong enough to include every certificated nurse throughout the British dominions, so that the Royal College of British Nursing may become the centre of all English-speaking nurses. In addition to Scotland and Ireland, Mr. Stanley has inquiries from Canada and Australia, where the wish exists to form branches of the College, so the outlook for the fulfilment of these great ideals is promising indeed.

Finally, Mr. Stanley expressed an earnest desire that a very large number of the nurses themselves should become members of the General Nursing Council through the Royal College of British Nursing. Parliament, he declared, would gladly give its support to such a policy. Everybody connected with the College had only one idea in mind, and that is to do each one his or her very best and utmost for the nursing profession and for the nurses who are working so courageously in the interests of the British Empire at the present time.

